# Population-based study in a rural area: methodology and challenges

**DOI:** 10.11606/S1518-8787.2018052000270

**Published:** 2018-09-13

**Authors:** Helen Gonçalves, Elaine Tomasi, Luciana Tovo-Rodrigues, Renata Moraes Bielemann, Adriana Kramer Fiala Machado, Ana Carolina Oliveira Ruivo, Caroline Cardozo Bortolotto, Gustavo Pêgas Jaeger, Mariana Otero Xavier, Mayra Pacheco Fernandes, Rafaela Costa Martins, Roberta Hirschmann, Thais Martins da Silva, Maria Cecília Formoso Assunção

**Affiliations:** I Universidade Federal de Pelotas . Faculdade de Medicina . Programa de Pós-Graduação em Epidemiologia. Pelotas , RS , Brasil; II Universidade Federal de Pelotas . Faculdade de Nutrição . Pelotas , RS , Brasil

**Keywords:** Health Surveys, methods, Data Collection, methods, Rural Population, Cross-Sectional Studies, Inquéritos Epidemiológicos, métodos, Coleta de Dados, métodos, População Rural, Estudos Transversais

## Abstract

**OBJECTIVE:**

To describe the planning, sampling, operational aspects of the field, and the sample obtained during a research conducted in a rural area, specifying and discussing the main logistical difficulties unique to these places and the solutions adopted.

**METHODS:**

We carried out a population-based, cross-sectional survey between January and June 2016, with a representative sample of the population aged 18 years or over living in the rural area of Pelotas (approximately 22,000 individuals), State of Rio Grande do Sul, Brazil. We collected demographic, socioeconomic, and health-related information, such as alcohol consumption, cigarette consumption, depressive symptoms, quality of diet, quality of life, physical activity, satisfaction with the health unit, overweight or obesity, and sleep problems.

**RESULTS:**

In the 720 domiciles sampled, 1,697 individuals were identified and 1,519 were interviewed (89.5%). The study initially drew 24 census tracts and proposed the visit to 42 households per tract; however, we need to adjust the method, such as decreasing the number of households per census tract (from 42 to 30) and identifying housing centers in each tract. The main reasons for these changes were difficulty accessing the area, large distances between households, misconceptions in the satellite data available (which did not fit the reality), and high cost of the field work.

**CONCLUSIONS:**

The previous detailed recognition of the research environment was crucial for decision making as the maps and territory had geographical inconsistencies. The strategies and techniques used in studies for the urban area are not applicable to the rural area given the outcomes observed in Pelotas. The decisions taken, keeping the methodological rigor, were essential to ensure the timely execution of the study with the financial resources available.

## RESUMO

**OBJETIVO:**

Descrever o planejamento, a amostragem, os aspectos operacionais do campo e a amostra obtida durante pesquisa realizada na zona rural, especificando e discutindo as principais dificuldades logísticas peculiares a esses locais e as soluções adotadas.

**MÉTODOS:**

Entre janeiro e junho de 2016, foi realizado inquérito transversal de base populacional, com amostra representativa da população com 18 anos de idade ou mais residente na zona rural de Pelotas (cerca de 22 mil), RS, Brasil. Foram coletadas informações demográficas, socioeconômicas e relacionadas à saúde, como consumo de bebidas alcoólicas, consumo de cigarros, sintomas depressivos, qualidade da alimentação, qualidade de vida, atividade física, satisfação com a unidade de saúde, excesso de peso ou obesidade e problemas do sono.

**RESULTADOS:**

Em 720 domicílios amostrados, 1.697 indivíduos foram identificados e 1.519 foram entrevistados (89,5%). O estudo, inicialmente, sorteou 24 setores e propôs-se a visitar 42 domicílios/setor, mas foram necessárias adequações metodológicas, especialmente a redução do número de domicílios por setor (de 42 para 30) e a identificação de núcleos habitacionais nos setores. As principais razões para as adequações foram dificuldade de acesso aos locais, grandes distâncias entre residências, equívocos nos dados geográficos disponíveis via satélite (não condiziam com a realidade) e alto custo.

**CONCLUSÕES:**

O prévio reconhecimento detalhado do ambiente de pesquisa foi fundamental para a tomada de decisão perante às inconsistências geográficas entre mapas e território. As estratégias e técnicas dos estudos na zona urbana não são aplicáveis à zona rural no que tange ao contexto observado em Pelotas. As medidas adotadas, mantendo o rigor metodológico, foram fundamentais para garantir a execução do estudo no tempo planejado e com os recursos financeiros disponíveis.

## INTRODUCTION

The Brazilian demographic occupation has changed since the 1950s ^1^ . While approximately 66% of the population lived in rural areas at the time ^2^ , approximately 85% of the population was concentrated in urban areas in 2010 ^3^ . Accelerated urbanization, especially between 1960 and 1980, has led to changes in the structure of occupation and work, with significant changes in the income generation, lifestyle, and health of the population ^4^ . Currently, public policies aimed at the Brazilian rural population seek to minimize investments, such as acquisition of technologies, and improve the quality of life ^5^ ^,^ ^6^ .

For effective health actions or the development of adequate public policies, we need to know the rural population and its specificities ^7^ ^,^ ^8^ . Few publications evaluate the general conditions or more than one health indicator of this population at the national level or in specific cities ^9^ . Most publications are focused on investigating some aspects of the health of rural workers ^9–12^ .

Brazilian surveys that have included the rural area in their sample, such as the National Household Sampling Survey (PNAD) ^13^ and the National Health Survey (PNS) ^14^ , have not published studies on health outcomes or reported the logistics used in the collection of the data in these regions – which is fundamental to guide and encourage new studies considering the logistic difficulties of these places.

Given the lack of population-based studies on the socioeconomic, demographic, and health characteristics of the adult population living in rural areas, the Post-Graduate Program in Epidemiology (PPGE) of the Universidade Federal de Pelotas carried out in 2015 its first population-based research in the rural area of the city of Pelotas. Since 1999, the PPGE has adopted the strategy of joint work with its master’s students in population-based studies, carried out until now in the urban area ^7^ . This strategy studies several health-related subjects in a single data collection, which allows students to be part of all phases of a research, from planning the research to writing reports and scientific articles, in addition to optimizing human and financial resources.

The objective of this study is to describe the planning, sampling, operational aspects of the field work, and the sample obtained during a research conducted in the rural area of Pelotas, and we specify and discuss the main logistical difficulties found in these places and the solutions we adopted.

## METHODS

### Coordination of the Study

The study had the support of the coordination of five professors of the PPGE, responsible for the planning and development of the field work and supervision of the nine master’s students. Professors and students held periodic meetings to solve doubts and evaluate the progress of the work.

### Design and Study Population

We carried out a population-based, cross-sectional survey between January and June 2016, with a representative sample of the population aged 18 years or over living in the rural area of Pelotas, State of Rio Grande do Sul, Brazil. The city is the third most populous of the state, with approximately 330,000 inhabitants; of these, approximately 22,000 live in the rural area ^3^ . This area has eight districts (Cascata, Cerrito Alegre, Colônia Z3, Monte Bonito, Quilombo, Rincão da Cruz, Santa Silvana, and Triunfo) and approximately 7% of the total population of the city.

The target population was identified in the households. We considered as domiciles all private households, which were characterized as where one or more persons live related by kinship ties (family), domestic dependency, or norms of coexistence. The definition of residents encompassed all individuals who usually lived in the rural area of Pelotas and who, at the reference date, were either present or absent for a period of no longer than 12 months, because they were traveling, hospitalized, in jail, or working ^15^ .

All residents in the mentioned age group were considered eligible for the study. We exclude those who were institutionalized or hospitalized during the entire period of data collection, those with some cognitive or mental disability unable to answer the questionnaire, and those who did not speak or understood Portuguese and did not have the help of caregivers or relatives.

The first step to develop the study – which is characterized as a broad project, with nine main research subjects – was to verify its viability. For that end, we contacted and investigated the possibility of receiving information and support from the (political and religious) leaders of the rural area of the city and from public entities [such as the Technical Assistance and Rural Extension Company (EMATER), the Brazilian Institute of Geography and Statistics (IBGE), the Municipal Health Department, and the City Department of Rural Development and its subprefectures]. All the contacts cooperated with the study and provided information on the following general characteristics of the population and districts: immigration, economy, health and social problems, road conditions and transportation, type of approach to be carried out with the population, and places to disseminate the study.

Each Master’s student developed their research project and calculated the sample size they needed, both to estimate the number of individuals required to study the prevalence and to examine possible associations. In all calculations, 10% were added to compensate for possible losses and refusals and 15% to calculate associations, in order to control possible confounding factors. Furthermore, the effect of sample design was considered according to each study subject, using multiple-stage sampling, as census tracts and, then, households would be randomly drawn. From these calculations, we defined the population to be studied. To this end, we considered the largest sample size needed for the analysis of outcomes with the expected precision, amounting to n = 2,016 adults.

After identifying the districts and their respective tracts, using the last Census ^3^ , we first drew 24 tracts among the 50 belonging to the rural districts ( Table 1 ). Based on the estimated sample size, we defined that 1,008 households should be visited considering the average of two adults per household, according to IBGE estimates ^3^ . We calculated the number of tracts (total and per district) from the number of permanent households in each district. This resulted in 42 households per tract. Subsequently, we decreased this number to 30 households per tract, amounting to 720 households for an estimated number of 1,440 individuals for the analyses of associations. This important change was needed given the logistical difficulties in identifying and accessing the households.

**Table 1:** Description of districts according to population size, number of permanent households, and number of tracts. Rural area of Pelotas, State of Rio Grande do Sul, Brazil, 2016.

District	Population*	Number of permanent households*	Total of number of tracts*	Number of selected tracts
Cascata	3,074	1,084	6	4
Cerrito Alegre	3,075	977	6	4
Colônia Z3	3,165	972	8	3
Monte Bonito	3,201	1,087	6	4
Quilombo	2,649	856	5	3
Rincão da Cruz	1,970	613	7	2
Santa Silvana	2,443	654	8	2
Triunfo	2,466	551	4	2

Total	22,043	6,794	50	24

* IBGE ^3^ (2010).

To identify these households, we used the software ‘Google Earth’, along with a virtual map of Pelotas provided by the local IBGE office. Using such instruments, we overlapped the geographical boundaries of the city, with the census tracts, over the satellite images provided by the software to count the eligible households. Subsequently, we identified housing centers, defined as clusters with at least five houses within a radius of one kilometer from its center.

We adopted the following procedure in each housing center to select the residences: starting in the center with the largest number of residences, we placed from its center a pointed object (such as a bottle) in a flat surface and rotated it to indicate the initial direction to the residences, thus ensuring randomness to the process. If the object pointed to a road branch, we always went to the right of the indicated direction. At the end of this path, we returned to the center and proceeded to the next road, located to the right of the first one, until we reached 30 households per tract. If we still could not reach this number, we headed towards the center of the second largest center of the tract, repeating the whole process. All eligible individuals identified in each sampled household were invited to participate in the study.

### Research Instruments

We developed and applied a household questionnaire and an individual questionnaire. In addition to these instruments, we performed anthropometry to collect data on weight, height, and waist circumference.

The household questionnaire was applied to a resident who could inform the composition, socioeconomic, and demographic data, and, when pertinent, the characteristics of the household and the rural property. The individual questionnaire aimed to collect information related to alcohol consumption, smoking, physical activity, sleep quality, depressive symptoms, food consumption and quality of the diet, quality of life, satisfaction with the basic unit and use of health services, general and abdominal obesity and other morbidities (self-reported), consumption and acquisition of medications, violence, consumption of mate ( *chimarrão* ), contact with pesticides, oral health, offspring, time living in the rural area, education level, and work or occupation. The instruments are available on website of the *Centro de Pesquisas Epidemiológicas* , at: http://epidemio-ufpel.org.br/site/content/mestrado/consorcio.php.

Trained interviewers collected the data, using tablets, with electronic versions of the questionnaires constructed using the website: https://projectredcap.org/ (REDCap – Research Eletronic Data Capture) *.* The digital version of the questionnaire and measure guide, as well as the scales and printed figures essential for some instruments, were also available during interviews.

These same interviewers were trained and standardized to collect anthropometric data. An inelastic measuring tape, WCS – Cardiomed ^®^ (with a length of 150 cm) graduated in millimeters and numbered every centimeter was used to measure waist circumference. A digital scale (Tanita ^®^ model Ironman BC558) with 150 kg limit and 100 g precision and a mountable adult anthropometer (Alturaexata ^®^ ) with a limit of 2.13 m and accuracy of 1 mm were used for the collection of weight and height, respectively.

### Training and selection of interviewers

The eligibility criteria for the interviewers were: being female (since, in our experience, women are more likely to be received by the population in home interviews), having complete high school, and availability of time. Characteristics such as previous experience in research, performance in the work, organization, and interpersonal relationship were also considered.

The first component of the training was the responsibility of the master’s students and took place in 12 shifts (morning, afternoon) with theoretical and practical exposition (role plays and tablets). In addition, we carried out a theoretical test, as well as the evaluation of attendance, participation, and interest. The candidates approved at this stage participated in the pilot study (second component of the training) carried out in the city of Arroio do Padre, next to Pelotas. This city was recently emancipated, and it is located between the districts of the rural area of Pelotas and still has characteristics similar to the area of the study. The pilot study aimed to evaluate the performance of each interviewer during a practical simulation of the field work. We hired the interviewers with the best performance during the training, written test, and pilot study.

In addition to these trainings to test and apply the questionnaires, the anthropometric data was standardized according to the methodology proposed by Habicht ^16^ . The master’s students were also standardized for these measures, as an alternative for eventual losses or faults of the team.

### Strategies to Disseminate the Research

Before and during data collection, the study had a widespread dissemination in the rural area and local media. We held meetings with district sub-prefects, with the City Departments of Rural and Health Development, as well as with workers of the basic health units to explain the research. We created and handed out information leaflets on the study to rural residents, we placed posters at strategic locations (bars, shops, churches, post offices, health units), and publicized the research using radios and local newspapers.

### Data Collection

Field work occurred in the seven days of the week. Transportation to the rural area was done by a microvan contracted for the study. On a daily basis, a master’s student on duty at the headquarters of the Program organized the material used in the field work, and at least two others accompanied the data collection in person.

The places to be visited were defined according to climatic conditions and the distance to be traveled, starting from the most distant places. The team carried a GPS (global positioning system) device and maps of the places of destination. The geographic coordinates of the households sampled and the time the interviewers were left in the residences were recorded. The daily working day varied according to location, harvesting periods, weather, and interview schedules, being, on average, seven hours.

At the household, one of the master’s students did the first contact with the residents’ presenting the research and inviting everyone to participate. At that moment, the household identification spreadsheet was filled in, which had the address, name of the residents, age, telephone number, interview shift, and who would answer the household questionnaire. After identification and scheduling, the household and individual instruments were applied to the eligible residents.

We considered as losses the individuals not found after at least three attempts at personal contact at different days and times, and we considered as refusals all individuals who did not agree to participate in the study.

Daily, after the team returned to the city, the tablets were collected for server synchronization and data transfer to the Stata program, version 14.0 (Stata Corporation, College Station, USA).

Weekly the master’s students performed a consistency analysis and notated the problems, which would be debated with the interviewers, and necessary corrections were made.

### Quality Control

In addition to the constant supervision of the daily planning of data collection and the work of the interviewers, 10% of the respondents were randomly drawn to participate in a quality control each week. A reduced questionnaire with 10 questions was applied to them by telephone to calculate the agreement between answers, using kappa statistics.

### Ethical and Financial Aspects

The project was approved by the Research Ethics Committee of the Faculdade de Medicina of the Universidade Federal de Pelotas (Process 1.363.979). All interviewees signed the informed consent.

Individuals identified with probable depression, according to the test score used, were given a list of services that have free psychological and psychiatric care and were invited to seek a local health service.

At the end of the study, a seminar open to the public was held for the presentation of the results of the research. Local authorities, and especially those from the rural area, were present and discussed the findings.

The study was funded by resources from the Commission for the Improvement of Higher Education Personnel (CAPES), small donations from local companies, and resources from the master’s students. Approximately US$20.00 was spent per resident.

### Data Analysis

The analyses were weighted to ensure the representativeness of the sample, according to the number of households sampled in relation to the total number of permanent households in each district. All statistical analyses were performed in the Stata 14.0 program using the survey ( *svy* ) command to consider the effect of sampling design. In this article, we present the prevalence and 95% confidence intervals of the main sociodemographic and economic characteristics of individuals and households.

## RESULTS

Table 1 describes the districts according to population size, number of permanent households, total number of census tracts, and selected tracts. Of the 22,000 inhabitants of the rural area of the city, according to the Census ^3^ , Monte Bonito was the district with the largest number of inhabitants and households. We selected two to four tracts in each district.

Of the 720 households sampled, 1,697 individuals were identified as eligible, 1,519 (89.5%) answered the questionnaire, and 178 (10.5%) were considered losses and refusals ( Table 2 ). Exclusions were due to physical (deafness without a translator at the household or cerebral palsy) or emotional disability (deep depression) and communication only in the Pomeranian dialect (without the presence of an interpreter at the household). Losses and refusals differed in relation to the variables of sex, age, and district of residence (p < 0.05).

**Table 2:** Characteristics of the sample, losses, and refusals of the study. Rural area of Pelotas, State of Rio Grande do Sul, Brazil, 2016.

Variable	Total eligible (%)	Sample interviewed (%)	Refusals (%)	Losses (%)	p*
Sex					< 0.001
Male	860 (50.7)	734 (48.3)	63 (69.7)	63 (72.5)	
Female	837 (49.3)	785 (51.7)	28 (30.3)	24 (27.5)	
Age (years)					0.010
18–24	206 (12.2)	174 (11.4)	11 (12.2)	21 (24.4)	
25–39	387 (22.9)	341 (22.6)	25 (27.3)	21 (24.7)	
40–59	658 (38.9)	593 (39.2)	31 (34.2)	34 (39.1)	
60–93	446 (26.0)	411 (26.8)	24 (26.3)	11 (11.8)	
District					0.001
Cascata	279 (16.1)	251 (16.2)	23 (24.5)	5 (5.5)	
Cerrito Alegre	266 (13.5)	245 (13.9)	10 (9.3)	11 (10.6)	
Colônia Z3	196 (13.6)	163 (12.7)	14 (17.9)	19 (25.1)	
Monte Bonito	257 (14.7)	232 (14.9)	5 (5.3)	20 (21.7)	
Quilombo	180 (10.9)	157 (10.6)	8 (8.9)	15 (17.2)	
Rincão da Cruz	134 (8.8)	120 (8.8)	6 (7.3)	8 (10.0)	
Santa Silvana	184 (10.7)	167 (10.9)	14 (15.0)	3 (3.3)	
Triunfo	201 (11.7)	184 (12.0)	11 (11.8)	6 (6.6)	

* Chi-square for the difference between sample and losses or refusals.

Most respondents were female, the age of the participants ranged from 18 to 93 years, and most individuals were aged 40 years or over. The median age and the interquartile range (values in parentheses) were 47 (28), 40 (27), and 44 (29) years between interviewees, losses, and refusals, respectively.

The average number of residents per household was 3.03 (SD = 1.5). Table 3 describes the characteristics of the households visited (n = 720). Only 6% of the households had six or more residents, more than half of the household heads had incomplete elementary education, almost half of the properties were supplied by water from a well in the property or spring, approximately 80% of the households had garbage collection performed by local garbage collection service, 72% of the households were built on owned land, and 88% of the sewage was drained to a septic tank.

**Table 3:** Characterization of the households of the sample (n = 716)*. Rural area of Pelotas, State of Rio Grande do Sul, Brazil, 2016.

**Variable**	**n**	**Proportion (95%CI)**
Number of residents		
Up to 2	303	42.0 (36.9–47.3)
3 to 5	370	52.2 (48.5–55.8)
6 or more	43	5.8 (3.3–10.1)
Education level of the family head		
No education	35	4.9 (3.4–7.1)
Incomplete elementary school	486	68.3 (61.5–74.4)
Complete elementary school/Incomplete high school	87	12.5 (10.1–15.5)
Complete high school/Incomplete higher education	73	9.9 (6.8–14.2)
Complete higher education or above	31	4.3 (2.6–7.0)
Supply of water		
General network	187	28.1 (15.2–45.9)
Well or spring in the property	350	47.7 (36.4–59.1)
Well or spring outside the property	143	19.4 (12.9–28.2)
Other	35	4.9 (3.1–7.6)
Piped water	695	96.9 (94.6–98.2)
Destination of the garbage		
Collected by the garbage collection service	558	78.1 (62.0–88.6)
Burned	134	18.6 (9.7–32.6)
Other	24	3.4 (1.8–6.3)
Land		
Owned	516	72.2 (65.7–77.8)
Borrowed	94	12.9 (9.7–17.0)
Possessed	53	7.6 (3.7–15.0)
Other	52	7.3 (5.0–10.5)
Sewage drainage		
Septic tank	621	88.3 (83.6–91.7)
General network	32	5.1 (2.2–11.4)
Ditch/Other	46	6.6 (4.7–9.3)
Activities developed		
Fishing	33	6.1 (-2.5–14.7)
Livestock	51	6.9 (4.2–9.6)
Plantation	119	16.4 (9.2–23.6)
More than one rural activity (fishing or livestock or plantation)	80	10.6 (4.6–16.6)
Non-rural activities	433	60.0 (48.2–71.9)

* 4 losses.

Table 4 summarizes the demographic, socioeconomic, and household characteristics of the sample, stratified by sex. Most respondents were white men and women, had European ancestry, and belonged to socioeconomic class C. Women had more years of education than men. Most men and women cohabited with a partner. More men were working at the time of the study than women, and most men and women worked in the rural area and carried out rural activities. Most were Evangelicals and Catholics, and more women reported having received a diagnosis of hypertension, as well as diabetes and high cholesterol. Men reported more contact with pesticides than women.

**Table 4:** Distribution of the sample of respondents according to demographic, socioeconomic, and household characteristics. Rural area of Pelotas, State of Rio Grande do Sul, Brazil, 2016.

**Variable**	**n (%)**	**Proportion (95%CI)**	

		**Men**	**Women**
Age (years)			
18–24	174 (11.5)	12.4 (10.4–14.6)	10.4 (8.4–12.8)
25–39	341 (22.4)	22.4 (18.9–26.4)	23.5 (19.4–28.1)
40–59	593 (39.0)	39.6 (36.4–42.9)	38.6 (35.8–41.6)
60–93	411 (27.1)	25.6 (21.8–30.0)	27.5 (24.0–31.3)
Race			
White	1,296 (85.3)	85.3 (79.2–89.1)	84.5 (77.6–89.5)
Black, brown, or yellow	223 (14.7)	14.7 (10.1–20.8)	15.5 (10.5–22.4)
Predominant ancestry ^a^			
European	913 (60.1)	61.9 (53.3–70.1)	56.9 (47.1–66.1)
Indigenous	27 (1.8)	2.0 (1.1–3.7)	1.7 (0.9–3.2)
African	50 (3.3)	2.4 (1.2–4.7)	4.2 (2.2–7.6)
Eastern	2 (0.1)	0.2 (0.0–1.2)	0.2 (0.0–0.0)
Mixed ^b^	463 (30.5)	29.7 (23.7–36.5)	32.4 (25.4–40.4)
Undefined classification	64 (4.2)	3.8 (2.3–6.1)	4.6 (3.2–6.7)
Socioeconomic level (ABEP) ^c^			
A or B	301 (20.0)	20.0 (14.7–26.6)	19.9 (14.7–26.4)
C	814 (54.2)	56.5 (50.8–62.0)	51.2 (45.5–56.9)
D or E	388 (25.8)	23.5 (18.1–29.9)	28.9 (22.8–35.8)
Education level			
No education	88 (5.8)	5.3 (3.0–9.3)	6.6 (5.1–8.4)
Incomplete elementary school	908 (60.2)	62.8 (54.8–70.2)	57.7 (50.5–6.5)
Complete elementary school or incomplete high school	233 (15.4)	16.6 (13.4–20.4)	14.2 (11.5–17.4)
Complete high school or incomplete higher education	213 (14.1)	12.8 (9.1–17.7)	15.1 (11.3–19.9)
Complete higher education or above	67 (4.4)	2.5 (1.4–4.2)	6.4 (4.2–9.6)
Living with a partner	1,076 (70.8)	70.0 (66.2–73.5)	71.8 (68.1–75.2)
Current work	906 (59.6)	73.4 (67.5–78.6)	46.3 (38.7–54.0)
Workplace ^d^			
Urban area	125 (13.8)	14.0 (8.4–22.4)	14.3 (8.0–24.2)
Rural area	720 (79.6)	78.4 (68.6–85.8)	80.9 (70.4–88.3)
Both	60 (6.6)	7.6 (5.1–11.3)	4.8 (2.7–8.5)
Occupation related to rural activity ^d^	509 (65.3)	67.7 (54.8–78.4)	61.8 (43.8–77.1)
Religion ^e^			
Catholic	599 (45.9)	48.2 (33.4–63.4)	46.4 (34.7–58.6)
Evangelical	595 (45.6)	43.8 (28.5–60.5)	44.9 (32.1–58.4)
Other	110 (8.4)	8.0 (5.2–12.0)	8.7 (6.3–11.8)
Hypertension ^f^	522 (34.4)	30.5 (26.0–35.3)	37.7 (33.6–42.1)
Diabetes ^f^	153 (10.1)	8.9 (6.9–11.3)	11.5 (9.5–14.2)
High cholesterol ^f^	291 (19.2)	16.5 (13.9–19.6)	22.3 (19.0–26.0)
Contact with pesticide (ever in life)	749 (49.3)	62.2 (52.0–71.5)	35.6 (27.1–45.3)

ABEP: *Associação Brasileira de Empresas de Pesquisa* (Brazilian Association of Research Companies)

^a^ Self-reported ancestry.

^b^ Two or more categories of ancestry.

^c^ Variable with most missing data.

^d^ Only for individuals who work.

^e^ Only for individuals who have a religion.

^f^ Medical diagnosis report.

## DISCUSSION

This was the first population-based household study carried out in a medium-sized city in Southern Brazil, with residents of a rural area, outlining relevant aspects of the health of these individuals.

Few published national and international studies have focused on the investigation of different health subjects with a large sample of rural residents. Almost all national population-based studies are carried out in the urban area. National surveys, such as PNS, PNAD, and LENAD ^13^ ^,^ ^14^ ^,^ ^17^ , which encompass rural areas, generally disclose prevalences of only a few health outcomes in the rural population, and they do not allow the identification of risk groups. This lack of rural studies can be understood in view of a number of difficult issues, especially given the logistics and financial costs to carry them out.

Commonly, the rural area of Brazil has unpaved roads and hard-to-reach places. A population-based study requires, besides the knowledge of the location, a series of strategies to reach the households and locate the residences with individuals within the properties. The available data, for example in Google Maps, does not allow buildings to be identified as households. The georeferenced images also do not always reflect the existence of geographic conditions to access the households; that is, the existence of streams, thicket, narrow or impassable roads, and interrupted bridges can only be verified in person. In addition, there are unexpected situations, such as the presence of loose animals on the roads or domestic animals in the households, which hinder the access.

In addition to the logistical difficulties mentioned above, which are probably common to other locations, other access barriers were identified in the rural area of Pelotas: lack of regular public transportation that covered most of the districts, households closed most of the day, resistance of residents in giving information on the first contact, inexistence or instability of telephone signal. It is also worth mentioning the feeling of insecurity in some locations because of isolation (without neighborhood and constant movement), violence (possibility of robberies or other losses), and the presence of drug trafficking sites (preventing the access to nearby residents or household).

All situations mentioned have repercussions on the financial cost and the time of execution of the study. The high cost of the study in Pelotas comes mainly from the repeated trips to districts and households far from the urban area, the hiring of private transportation, and the difficulties in matching the time when the residents were available for data collection and when interviewers could visit them. In addition, the wear of the vehicle from the distance traveled daily – included in the total amount –, the need to work on the weekends, the workday restricted to the brightness of the day and limited to a shift of six consecutive hours – obeying labor laws –, and the available time of the driver also contributed to this cost.

According to the estimates of cost and duration of the research, we needed to recognize that we could not carry out the prior identification of the households in each tract, as it is done in research studies in urban areas. We attempted to execute this process with the adoption of several procedures, such as the printing of maps with the residences located by satellite and the purchase of devices that had a global positioning system (GPS) for the recognition and location of households using specific geographical coordinates. However, the geographic coordinates often did not refer specifically to the households, but to other points in the property to be visited (e.g., center of a large farm, dam, or trade). These findings led to the need to review the planning for household selection. Therefore, the new strategy consisted of dividing the census tracts into housing centers, with the aid of overlapping aerial images (via Google Earth) and geographic delimitations, as described in the methods section.

Another important modification was the reduction of households to be sampled, without changing the number of tracts selected (from 42 households per tract to 30 households per tract). This, however, reduced the statistical power for some specific associations studied, which are highlighted in the articles of this supplement.

Even with all the difficulties pointed out, the study was successful with a high response rate (89.5%). However, we may have had sample selection bias (“asphalt bias”) ^18^ , as the selection of the housing center with the largest cluster of households may have led to the choice of households closer to roads and highways. Furthermore, previous studies have evaluated the pros and cons of the randomization strategy used, called random walk (selection of households from the direction pointed by an object, for the follow-up of the sampling throughout the neighborhood) ^19^ ^,^ ^20^ *.* There are publications that both point to sample selection bias in this process and report that there is little variation in the prevalence estimate when this process is adopted ^19^ ^,^ ^20^ . There is also the possibility of other biases, including those of measurement and reverse causality (common in cross-sectional studies). The impossibility of determining cause and effect and the fact that certain exposures and outcomes may be more common among rural residents means that the results should be interpreted with caution. However, each article resulting from this study exposes how it considered or minimized potential biases in its analyses.

The importance of a detailed recognition of the research environment was evident in this study. Previous recognition was fundamental for decision-making given the geographical inconsistencies between maps and territory. The strategies and techniques of studies in the urban area are not applicable to the rural area regarding the context observed in Pelotas. The measures adopted, keeping the methodological rigor, were essential to ensure the timely execution of the study with the financial resources available.
